# The Effect of Fullerenol C_60_(OH)_36_ on the Antioxidant Defense System in Erythrocytes

**DOI:** 10.3390/ijms23010119

**Published:** 2021-12-23

**Authors:** Jacek Grebowski, Paulina Kazmierska-Grebowska, Natalia Cichon, Piotr Piotrowski, Grzegorz Litwinienko

**Affiliations:** 1 Department of Molecular Biophysics, Faculty of Biology and Environmental Protection, University of Lodz, Pomorska 141/143, 90-236 Lodz, Poland; 2 The Military Medical Training Center, 6-Sierpnia 92, 90-646 Lodz, Poland; 3 Department of Neurobiology, Faculty of Biology and Environmental Protection, University of Lodz, Pomorska 141/143, 90-236 Lodz, Poland; paulina.kazmierska@biol.uni.lodz.pl; 4 Biohazard Prevention Centre, Faculty of Biology and Environmental Protection, University of Lodz, Pomorska 141/143, 90-236 Lodz, Poland; natalia.cichon@biol.uni.lodz.pl; 5 Faculty of Chemistry, University of Warsaw, Pasteura 1, 02-093 Warsaw, Poland; ppiotrowski@chem.uw.edu.pl (P.P.); litwin@chem.uw.edu.pl (G.L.)

**Keywords:** fullerenol, antioxidant, water-soluble derivatives of fullerenes, oxidative stress, erythrocytes

## Abstract

*Background*: Fullerenols (water-soluble derivatives of fullerenes), such as C_60_(OH)_36_, are biocompatible molecules with a high ability to scavenge reactive oxygen species (ROS), but the mechanism of their antioxidant action and cooperation with endogenous redox machinery remains unrecognized. Fullerenols rapidly distribute through blood cells; therefore, we investigated the effect of C_60_(OH)_36_ on the antioxidant defense system in erythrocytes during their prolonged incubation. *Methods*: Human erythrocytes were treated with fullerenol at concentrations of 50–150 µg/mL, incubated for 3 and 48 h at 37 °C, and then hemolyzed. The level of oxidative stress was determined by examining the level of thiol groups, the activity of antioxidant enzymes (catalase, glutathione peroxidase, glutathione reductase, and glutathione transferase), and by measuring erythrocyte microviscosity. *Results*: The level of thiol groups in stored erythrocytes decreased; however, in the presence of higher concentrations of C_60_(OH)_36_ (100 and 150 µg/mL), the level of -SH groups increased compared to the control. Extending the incubation to 48 h caused a decrease in antioxidant enzyme activity, but the addition of fullerenol, especially at higher concentrations (100–150 µg/mL), increased its activity. We observed that C_60_(OH)_36_ had no effect on the microviscosity of the interior of the erythrocytes. *Conclusions*: In conclusion, our results indicated that water-soluble C_60_(OH)_36_ has antioxidant potential and efficiently supports the enzymatic antioxidant system within the cell. These effects are probably related to the direct interaction of C_60_(OH)_36_ with the enzyme that causes its structural changes.

## 1. Introduction

Oxidative stress is a consequence of an increase in the level of reactive oxygen species (ROS) generated by physical or chemical agents, such as ionizing and UV radiation, ultrasound, peroxides, heavy metal ions, herbicides, and several drugs [1]. An additional endogenous source of ROS is intracellular respiration and other metabolic processes [2]. Organisms have developed several protective systems that include antioxidants and enzymes responsible for the redox balance [3,4], with glutathione transferase (GST), glutathione peroxidase (GPx), glutathione reductase (GSR), and catalase (CAT) as examples of the most important enzymes acting in healthy cells [5], and also during carcinogenesis [6]. Glutathione is a key molecule responsible for the deactivation of radicals and regulation of the activity of the above enzymes; therefore, any disruption of redox homeostasis connected with oxidative stress can be evaluated on the basis of the activity of the glutathione-dependent enzymes [7].

Fullerenes and their derivatives are considered effective free radical scavengers, including ROS, based on the large number of conjugated double *π* bonds [8]. Their water-soluble derivatives C_60_(OH)_n_ (fullerenols) have recently been the subject of numerous investigations regarding the radioprotective and antioxidant properties in biological and biomimetic systems [9,10,11,12,13,14,15,16,17]. Covalently attached hydroxyl groups play a crucial role for the localization of fullerenol and its accessibility for ROS, whereas the mechanism of antioxidant action of C_60_(OH)_n_ depends on the nature of ROS [18]. Apart from ^1^O_2_, O_2_^•−^ and ^•^OH, and peroxyl radicals, C_60_(OH)_n_ are potential scavengers of reactive nitrogen species, such as NO (a precursor of highly reactive peroxynitrite), lipid peroxides, and superoxyls [8,13,19,20,21]. Highly hydroxylated fullerenols inhibit the peroxidation of unsaturated lipids, and in combination with pentamethyl hydroxychromane (PMHC, an analogue of α -tocopherol) a hyper-synergy is observed at pH 7 and 10 [17]. A combination of the high polarity (bioavailability) of C_60_(OH)_36_ with its ability to effectively trap radicals makes these molecules potentially good inhibitors of oxidative damage. Due to its convenient localization at the lipid/water interface, fullerenol C_60_(OH)_36_ should exhibit a high degree of selectivity. We previously demonstrated that fullerenol C_60_(OH)_36_ is a strong antioxidant that inhibits the lipid peroxidation process in isolated human erythrocyte membranes (TBARS) and inhibits the peroxidation process in various model systems, including liposomes and micelles [17]. In addition to radical reactions, C_60_(OH)_n_ can also remove metal ions from the system by forming insoluble cross-linked metal –hydroxyfullerene polymers [22].

Highly hydroxylated fullerenols undergo rapid distribution within blood cells [17,23,24,25], and we decided to use this feature for biological applications of C_60_(OH)_36_ [8,26]. We reported that C_60_(OH)_36_ interacts with erythrocyte membrane proteins through the available protein -SH groups [27]. Lichota et al. demonstrated the ability of C_60_(OH)_36_ to enter cells and the influence of mitochondrial membrane potential [28]. Furthermore, the latest results of Lee et al. suggested that C_60_(OH)_36_ acts against particulate matter-induced cytotoxicity via ROS scavenging and anti-inflammatory mechanisms, and the maintenance of the expression of barrier proteins [29].

Mature human erythrocytes constitute a convenient research model to study oxidative damage during prolonged incubation, which triggers ROS-mediated damage [30,31,32,33,34]. The source of ROS in erythrocytes is hemoglobin, which undergoes autoxidation to methemoglobin producing O_2_^•−^ [30]. Both products are dangerous for cells. Superoxide is easily transformed into the potent oxidant H_2_O_2_. ROS can be detoxicated, and MetHb is reduced to ferro-Hb. Reduced coenzymes play an important role in the antioxidant defense of erythrocytes. NADH produced by the glycolytic pathway is the main reductant of MetHb to ferro-Hb. NADPH produced by the hexose monophosphate shunt is a co-substrate for glutathione reductase and plays an important role in maintaining the catalytic activity of catalase [30]. We have previously indicated that, along with the prolonged incubation time, the production of reducing equivalents of NADH and NADPH (coenzymes for enzymatic antioxidant defense) decreases gradually. After a 48 h incubation time in PBS, a depletion of low-molecular energy compounds, such as ATP and NADPH, occurs and structural changes appear. Consequently, the efficiency of the erythrocyte defense system declines, which results in oxidative damage [30]. Erythrocytes are extremely sensitive to oxidative damage due to the lack of cellular organelles that would be responsible for replacing damaged components. Thus, oxidative damage can induce permanent defects that ultimately lead to hemolysis in vitro [30]. Taking into account the above, this study aimed to investigate the effect of fullerenol C_60_(OH)_36_ as a potential free radical scavenger on the antioxidant system of erythrocytes in terms of prolonged incubation.

## 2. Results

### 2.1. Total Concentration of -SH Groups in Erythrocytes Incubated with C_60_(OH)_36_

The effect of C_60_(OH)_36_ on the level of antioxidant damage to proteins was analyzed by measuring the concentration of the -SH groups. This was determined on the basis of the P parameter calculated as previously described [35]. The concentration of -SH groups was obtained from the calibration curve. Summarized, after 3 h incubation with C_60_(OH)_36_, the level of -SH groups was still the same as in the control sample. After 48 h of incubation, the concentration of -SH groups decreased in both control and analyzed samples; however, in the presence of higher concentrations of C_60_(OH)_36_ (100 and 150 µg/mL), -SH groups disappeared slower compared to the control (for details. see Table 1).

### 2.2. Activity of Enzymes in Erythrocytes Incubated with C_60_(OH)_36_

**Figure 1:**
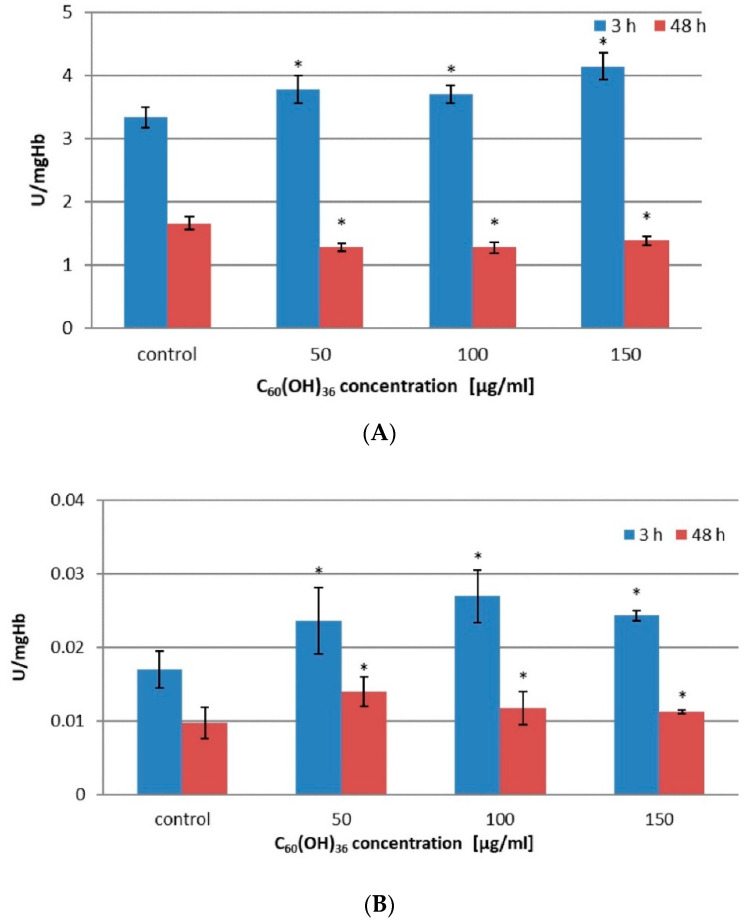
The effect of C_60_(OH)_36_ on the activity of catalase (**A**) and glutathione peroxidase (**B**) converted to 1 mg of hemoglobin protein. Measurements were made after 3 and 48 h of incubation of erythrocytes with C_60_(OH)_36_ at concentrations of 0, 50, 100, and 150 µg/mL at 37 °C. The bars represent the mean values ± SD for 3 independent measurements. * Statistically significant values in relation to controls after 3 h and 48 h incubation at *p* < 0.05.

The effect of C_60_(OH)_36_ on catalase activity (CAT) was assessed in 2% hematocrit incubated with C_60_(OH)_36_ for 3 and 48 h at 37 °C. The final concentration of C_60_(OH)_36_ in suspension ranged from 0 to 150 µg/mL. The results obtained are presented in Figure 1.

For concentrations above 50–150 µg/mL, C_60_(OH)_36_ significantly increased CAT activity after 3 h of incubation compared to control (*p* < 0.05). After extending the incubation time to 48 h, the activity of CAT in the same concentration range was significantly reduced compared to the samples not treated with C_60_(OH)_36_ (*p* < 0.05) (for details, see Table 2 and Figure 1A).

Figure 1B presents changes in glutathione peroxidase (GPx) activity. A statistically significant increase in enzyme activity was observed after 3 h of incubation in 100 µg/mL and 150 µg/mL C_60_(OH)_36_ compared to the control (*p* < 0.05). On the other hand, prolonging the incubation time to 48 h resulted in a statistically significant increase in enzyme activity when erythrocytes were incubated in C_60_(OH)_36_ in a concentration range of 50–150 µg/mL in comparison to the control (*p* < 0.05) (for details, see Table 2).

**Figure 2:**
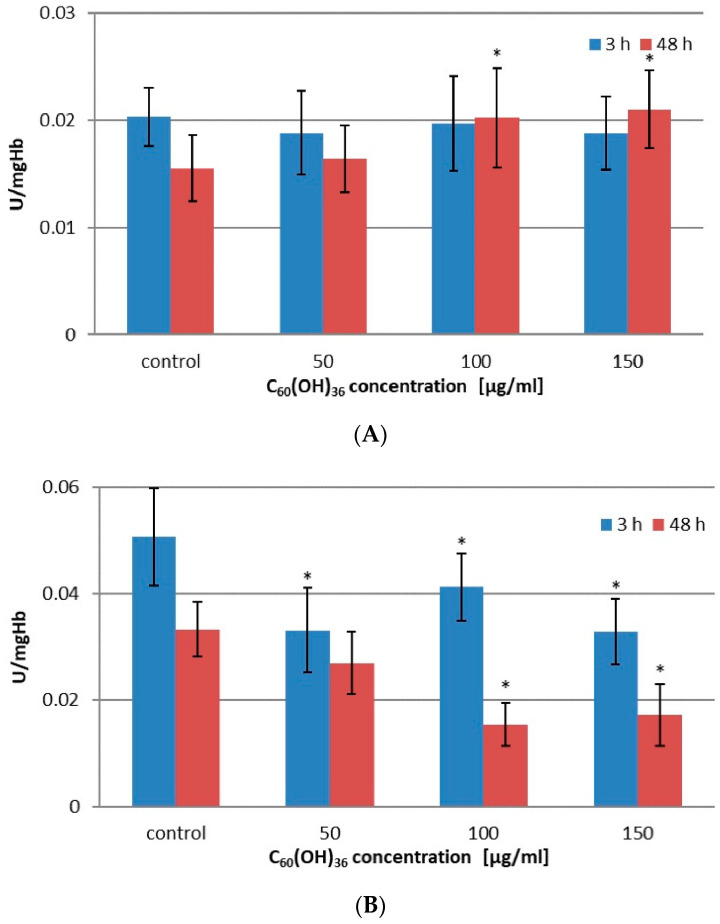
(**A**) Effect of C_60_(OH)_36_ on the activity of glutathione reductase. Measurements were made after 3 and 48 h of incubation with C_60_(OH)_36_ at a concentration of 0, 50, 100, and 150 µg/mL at 37 °C. The bars represent the mean values ± SD for 3 independent measurements. * Statistically significant values relative to controls after 48 h of incubation at *p* < 0.05. (**B**) Effect of C_60_(OH)_36_ on the activity of glutathione transferase. Measurements were made after 3 and 48 h of incubation with C_60_(OH)_36_ at a concentration of 0, 50, 100, and 150 µg/mL at 37 °C. The bars represent the mean values ± SD for 3 independent measurements. * Statistically significant values relative to controls after 3 and 48 h of incubation at *p* < 0.05.

The changes in activity of two other enzymes belonging to the glutathione family, glutathione reductase (GSR) and glutathione transferase (GST), are presented in Figure 2 and Table 3.

After 3 h of incubation, no statistically significant changes were observed compared to control; however, the extension of the incubation time to 48 h in C_60_(OH)_36_ at concentrations of 100 µg/mL and 150 µg/mL (*p* < 0.05) resulted in an increase in GSR enzymatic activity (for details, see Table 3 and Figure 2A). After 48 h of incubation in C_60_(OH)_36_ at concentrations of 100 µg/mL and 150 µg/mL, we saw a significant increase in GSR activity compared to the control (for details see Table 3 and Figure 2A). 

The last enzyme, also belonging to the glutathione family, was glutathione transferase (GST) (see Figure 2B and Table 3). The behavior of this enzyme is different from other free enzymes, because after incubation (3 and after 48 h) and hemolysis, we noticed a decrease in activity compared to the control sample (without C_60_(OH)_36_). Specifically, a 3 h incubation with C_60_(OH)_36_ at concentrations of 50 µg/mL, 100 µg/mL, and 150 µg/mL (*p* < 0.05) resulted in a decrease of glutathione transferase activity compared to the control, but long incubation caused a decrease in GST activity (with respect to the control, *p* < 0.05) for 100 µg/mL and 150 µg/mL C_60_(OH)_36_ (for details, see Table 3).

### 2.3. The Effect of C_60_(OH)_36_ on Erythrocyte Microviscosity

Measurements were made using the Tempamine spin tracer, which penetrates relatively easily through the plasma membrane into the erythrocyte interior [36], and the method compares the value of the rotational correlation time of Tempamine inside the cell, referring to a solution with known viscosity [37]. The effect of C_60_(OH)_36_ on the microviscosity of the interior of the erythrocyte was assessed in 2% hematocrit incubated with C_60_(OH)_36_ for 3 and 48 h at 37 °C. The final concentrations in suspension were 0, 50, 100, and 150 µg/mL. The results obtained are shown in Table 4, indicating that C_60_(OH)_36_ has no effect on the microviscosity of the interior of the erythrocytes. No statistically significant changes in microviscosity were observed after 3 h and after 48 h.

## 3. Discussion

In our experiments, we tested the enzyme activity in erythrocytes subjected to oxidative stress induced by prolonged incubation, in the presence of C_60_(OH)_36_. Fullerenes are an important class of nanomaterials with a wide potential applicability in biomedical sciences [8], and their water-soluble derivatives (fullerenols) are promising candidates for use as antioxidants in biological systems [10,11,12,13,14,15,16,17]. C_60_(OH)_36_ prevents ROS cell damage, but also can scavenge NO [13,20,21,38].

Reduced glutathione (GSH) is a primary intracellular source of thiol groups and an important hydrophilic reducing agent that protects cells from electrophiles and free radicals due to the nucleophilicity of -SH groups and reaction with oxidants [39]. In addition to the direct reaction with ROS, glutathione can sacrificially regenerate damaged macromolecules [7]. The results presented in Table 1 clearly demonstrate that prolonged incubation of erythrocytes causes a general reduction in -SH groups (control conditions). This is most likely an effect of oxidative stress, a decrease in ATP levels, and alterations in membrane structure and integrity, resulting in altered ion transport and changed erythrocyte shape [40]. We have observed that incubation in C_60_(OH)_36_ for 3 h had no effect on the level of the -SH groups; however, a prolonged 48 h incubation with 100 or 150 µg/mL C_60_(OH)_36_ resulted in a significant increase in the levels of the -SH groups in erythrocytes (32% and 41%, respectively). These results, indicating that C_60_(OH)_36_ used in relatively high concentrations could protect -SH groups against oxidation, are consistent with our previous findings [35], where we reported that C_60_(OH)_36_ significantly protected -SH groups from radiation-induced oxidation, and this protection was due to ROS removal by fullerenol. 

**Figure 3:**
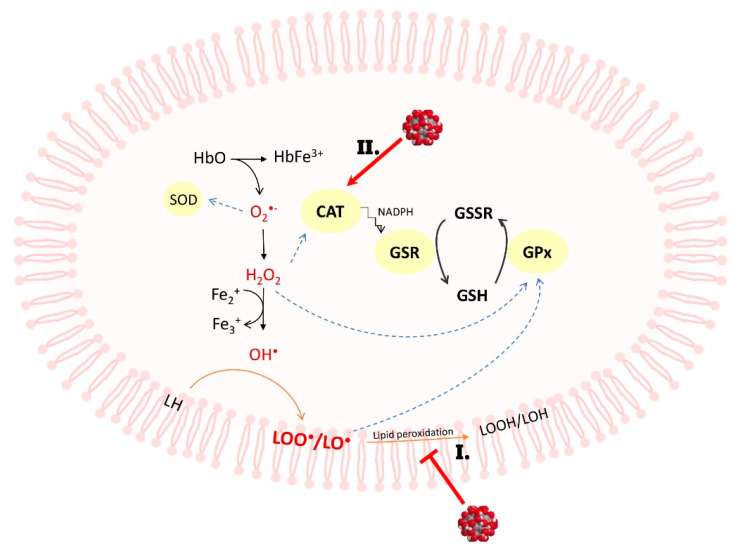
(**I**) Direct interaction with the membrane and radical scavenging of radicals by C_60_(OH)_36_ [17,24,35] and (**II**) increasing the activity of antioxidant enzymes such as GSR activity, resulting in an increase in the total pool of GSH, which is a cofactor of GPx, which reduced the formation of lipid peroxides. Therefore, the reduction in hemolysis observed in our previous work could be the result of both processes [35].

In our experiments, the activity of CAT was enhanced (up to 24%) in the presence of 50–150 µg/mL C_60_(OH)_36_ after 3 h of incubation, but after 48 h, the activity of catalase decreased by approximately 33% (Figure 1A, Table 2). We propose that C_60_(OH)_36_ might act as an allosteric effector for CAT, changing the protein structure. Enzymatic conformational changes induced by C_60_(OH)_36_ during short-term incubation could increase CAT activity, but prolonged incubation could cause accelerated oxidation of that enzyme [41]. Our findings partly support previously reported observation reported by Djordjevic et al. [21] that fullerenol protects CAT against the changes induced by NO radicals. The decrease in CAT activity after 48 h can cause the dissociation of NADPH [42], further used by GSR during the reaction to recover GSH from GSSG. An argument in favor of this hypothesis is the increase in the amount of GSH after 48 h of incubation with C_60_(OH)_36_ (100–150 µg/mL). Fullerenol C_60_(OH)_36_ increases GSR activity, resulting in an increase in the overall pool of GSH, which is a cofactor of GPx (Figure 3).

We noticed that a 3 h treatment with C_60_(OH)_36_ (100 and 150 µg/mL) significantly increased GPx activity, whereas a prolonged incubation (48 h) caused a decrease in GPx activity, however, this activity is still higher than in control samples (for details, see Table 2, Figure 1B). Because the effect of protection of GPx was not concentration dependent and after 3 h of incubation with C_60_(OH)_36_ the activity of CAT and GPx increased, we suppose that the observed effect can be assigned to the interactions of C_60_(OH)_36_ with the enzyme subunits. GPx is a protein that reduces lipid peroxidation, therefore preventing hemolysis, and this explains why in our previous experiments [24] we observed a reduction in hemolysis and potassium leakage after the use of C_60_(OH)_36_

The last enzyme studied in our work was GST. Human erythrocytes contain three GST isoenzymes: rho (ρ), sigma (σ), and theta (θ); the ρ isoenzyme belongs to the Pi (*π*) class, which shows 95% activity against the commonly used CDNB substrate [43]. This enzyme is sensitive to oxidative stress due to the presence of cysteine residues at positions 14, 47, 101, and 169 at the N terminus, which are susceptible to oxidation by ROS, such as H_2_O_2_ and HClO. Modification of the cysteine residue at position 47, located near the active center, caused a decrease in GST activity [44,45]. In our experiments, we noticed an inhibition effect of fullerenol. Shorter treatment time resulted in reduced GST activity by approximately 35%, regardless of the concentration of C_60_(OH)_36_, whereas for a longer time of incubation, a decrease in enzyme activity was approximately 55% (100 and 150 µg/mL C_60_(OH)_36_).

In the present study, we also examined the microviscosity of the interior of the erythrocyte in the presence and absence of C_60_(OH)_36_. Aging of erythrocytes is accompanied by an increase in the cell microviscosity of cells [46] because of a decrease in intracellular electrolyte concentration, a lower water content, and a reduction in cell diameter, volume, and surface area [32,47]. The cytoplasmic microviscosity of the interior of erythrocytes is determined mainly by the concentration of hemoglobin, but also depends on the water content of the cell [48]. Viscosity is regulated by the intracellular and extracellular amount of Na^+^ and K^+^ ions, which affects the cell volume [49]. The results collected in Table 4 do not show visible changes in erythrocyte microviscosity, and we can explain this lack of measurable changes as the effect of two opposing processes. First, C_60_(OH)_36_ causes a decrease in Na^+^/K^+^-ATPase activity, which could lead to ion imbalance and hemolysis [50,51,52]. On the other hand, hemolysis induced by prolonged incubation is inhibited by retention of hemoglobin within the erythrocyte [24]. Thus, both processes contribute to the apparent effect of no visible changes in microviscosity.

## 4. Materials and Methods

### 4.1. Synthesis of Fullerenol

Fullerenol (polyhydroxyfullerene) C_60_(OH)_36_ was synthesized by the solvent-free method proposed by Wang et al. [53], and, in a modified version was described in the previous paper of Grebowski et al. [50]. Fullerenol was prepared by grinding pristine fullerene C_60_ with NaOH and 30% hydrogen peroxide in a glass mortar in air at room temperature for 25 min (Wang et al. recommended 15 min), then the additional incubation of the reaction mixture at 60 °C (water bath) was carried out for 20 min. The crude mixture was dissolved in 50 mL of deionized water and hydrolysis was performed for 24 h. Fullerenol was precipitated with methanol, resolubilized with water, and traces of NaOH were removed by passing the solution over the ion exchange resin (Amberlit MB-20). The structure of the obtained hydroxyl derivative of fullerene C_60_ was confirmed by IR spectrophotometry (EXUS FT-IR spectrometer, Thermo Fisher Scientific, Waltham, MA, USA), ^1^H-NMR (Bruker Avance III 600 MHz, Bruker Corp., Billerica, MA, USA), ^13^C-NMR (Varian Gemini 200 MHz, Varian Inc., Palo Alto, CA, USA), MALDI-TOF/TOF mass spectrometry (Axima Performance Mass Spectrometer, Shimadzu Biotech, Kyoto, Japan) as described previously [35].

### 4.2. Erythrocyte Isolation

Blood from the leukocyte-platelet coagulum, taken from the Regional Center for Blood Donation and Transfusion in Lodz, was centrifuged at 4 °C in a horizontal rotor centrifuge for 5 min at 3500 rpm, with the plasma and leukocyte layer removed. Subsequently, the obtained erythrocytes were washed three times with cooled PBS pH 7.4 (145 mM NaCl, 10 mM phosphosodium) and centrifuged under the same conditions. After obtaining erythrocyte suspensions, the hematocrit was determined by the microcapillary method, and for further studies, the suspension was diluted with a PBS solution to a hematocrit of 2%.

### 4.3. Hemolysate Preparation

Purified erythrocytes were hemolyzed with distilled water. Hemolysate for the determination of CAT activity was prepared by adding 1 mL of water to 0.5 mL of erythrocyte suspension with a hematocrit of 2%, and thoroughly mixed and placed in the freezer for 30 min. The samples were then mixed and centrifuged at 7000 rpm for 5 min. Hemolysate for the determination of GSH-GPx, GR, and GST activities was prepared by centrifuging 0.5 mL of erythrocyte suspension with a hematocrit of 2% at 7000 rpm, collecting 0.48 mL of the solution, and adding 0.5 mL of water. The samples were then thoroughly mixed and placed in the freezer for 15 min, then at room temperature for 10 min, and centrifuged at 7000 rpm for 5 min. The hemolysates obtained were frozen at −70 °C.

### 4.4. Determination of Hemoglobin Concentration in Hemolysate Using the Drabkin Method

The hemoglobin concentration was determined in 96-well plates. To 280 µL of Drabkin’s reagent (0.03% K_3_[Fe(CN)_6_], 0.1% NaHCO_3_, 0.005% KCN), 20 µL of hemolysate (study sample), and 20 µL of distilled water (blank) were added. The absorbance at λ = 541 nm was measured.

### 4.5. Fullerenol Treatment of Erythorocytes 

A 5 mg/mL solution of C_60_(OH)_36_ in PBS was added to a 2% suspension of erythrocytes in PBS at a final concentration of 50, 100, and 150 µg/mL, respectively, and thoroughly mixed. The samples were incubated at 37 °C. The determinations were made after 3 and 48 h of incubation.

### 4.6. Identification of Erythrocyte Microviscosity

**Figure 4:**
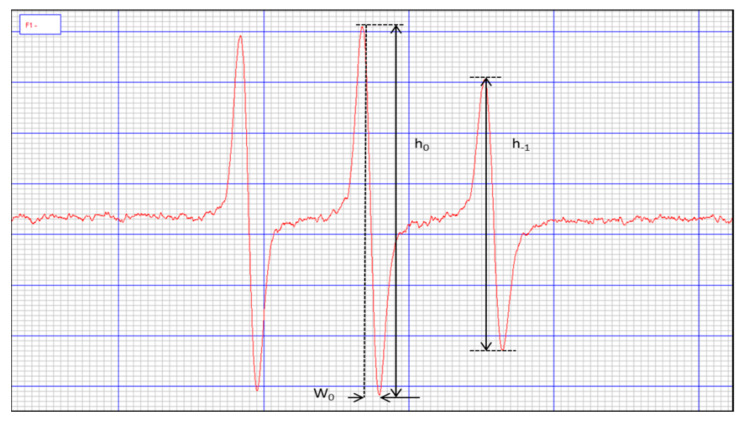
View of the Tempamine marker.

A 50% hematocrit was prepared by centrifuging 1.6 mL of the incubated erythrocyte suspension and collecting 1.54 mL of the supernatant. To the remaining 60 µL, 0.6 µL Tempamine (spin tracer: 10 mM of Tempamine dissolved in PBS) was added and stirred for 30 min at room temperature. The samples were then washed with 1 mL of 80 mM potassium ferricyanide and centrifuged, and the EPR spectra of the samples (see Figure 4), as well as the tracer spectrum in PBS, were determined. The rotational correlation time of the tracer was calculated from the formula:

$$\tau_{R}=kW_{0}\left(\sqrt{\frac{h_{0}}{h_{-1}}}-1\right)$$

where:

*k* = 6.5 × 10^−9^ s/mT
*W*_0_ = width of the spectral center line
*h*_0_ = height of the spectral midfield line
*h*_–1_ = height of the highfield line of the spectrum

The viscosity of the interior of the erythrocyte was calculated from the following equation:

$$\frac{\tau_{R}}{\tau_{B}}=\frac{\eta_{R}}{\eta_{B}}$$

where:

*τ_R_* = rotational correlation time of the tracer inside erythrocytes
*τ_B_* = rotational correlation time of the tracer dissolved in a solution of known viscosity
*η_R_* = erythrocyte inner viscosity
*η_B_* = viscosity of known solution

### 4.7. Total Concentration of -SH Groups in Erythrocytes

Incubated erythrocytes with 2% hematocrit were diluted to a hematocrit of 0.75% with a final volume of 160 µL. Then, 0.45 µL of tracer was added so that the final concentration in the sample was 80 µmol. After 3 min of incubation with the tag, EPR spectra were obtained similarly as described previously [35].

### 4.8. Determination of Catalase Activity

To a spectrophotometric cuvette, 3 mL of 0.018 M hydrogen peroxide in 0.05 M phosphate buffer (pH 7.0) at 25 °C was added so that the absorbance measured at 240 nm was approximately 1. Then 15 µL of hemolysate was added, and the decrease in absorbance at λ = 240 nm was measured for 1 min. The activity was referred to 1 mg of hemoglobin protein.

### 4.9. Determination of Glutathione Peroxidase Activity

Glutathione peroxidase activity was determined by an indirect method. In the spectrophotometer cuvette, 250 µL of phosphate buffer (0.1 M pH 7.0 with 0.1 mM EDTA) equilibrated at 37 °C, 50 µL of glutathione reductase (2.4 units/mL), 50 µL of 10 mM GSH, 50 µL of 1.5 mM NADPH in a 0.1% NaHCO_3_ solution, 50 µL of hemolysate to which 2 µL of 100 mM sodium azide was added to block catalase, and 2 µL of a mixture of 60 mM K_3_Fe(CN)_6_ and 48 mM KCN solutions were added to convert hemoglobin to cyanmethemoglobin. The reaction was initiated by adding 50 µL of 12 mM t-butyl hydroperoxide. The decrease in absorbance at λ = 340 nm was measured for 3 min. The activity was referred to 1 mg of hemoglobin protein.

### 4.10. Determination of Glutathione Transferase Activity

Eight hundred fifty µL of phosphate buffer (0.1 M pH 6.5) equilibrated at 37 °C was added to the spectrophotometer cuvette followed by 50 µL of 0.02 GSH solution, 50 µL of CDNB solution (0.02 M solution of 1-chloro-2,4-dinitrobenzene dissolved in ethanol), and 50 µL of hemolysate. The absorbance at λ = 340 nm was measured for 1 min. The activity was referred to 1 mg of hemoglobin protein.

### 4.11. Determination of Glutathione Reductase Activity

One thousand µL of 2.2 mmol/L oxidized glutathione solution at 37 °C was added to a spectrophotometer cuvette followed by 40 µL of hemolysate. It was thoroughly mixed and 200 µL of 0.17 mmol/L of NADPH was added. The decrease in absorbance was measured at λ = 340 nm for 2 min. The activity was referred to 1 mg of hemoglobin protein.

### 4.12. Statistical Analysis

All experiments were carried out 3 to 8 times. Values were expressed as mean ± standard deviation (SD). Data were analyzed by one-way analysis of variance (ANOVA), followed by Tukey’s post-hoc test, all using GraphPad 4.0 software (La Jolla, CA, USA).

## 5. Conclusions

The results presented here reveal that C_60_(OH)_36_ can modulate the redox balance of human erythrocytes subjected to oxidative stress induced during a prolonged incubation in PBS at 37 °C. Apart from direct antioxidant action, fullerenol interacts with the antioxidant enzymes and stimulates them to more efficient and longer activity. A 3 and 48 h treatment with C_60_(OH)_36_ (50–150 µg/mL) maintains the proper function of the antioxidant system (CAT and GPx). However, we hypothesize that changes in its activity are not caused directly by the involvement of ROS, but conformational changes induced by C_60_(OH)_36_ itself. These effects are related to the concentration of C_60_(OH)_36_ and to the kind of parameters examined that characterize cell damage.

## Figures and Tables

**Table 1:** Total concentration of -SH groups in erythrocytes incubated at 37 °C with C_60_(OH)_36_.

C_60_(OH)_36_ Concentration [µg/mL]	Incubation Time					
	3 h			48 h		
	Parameter P	SD	Thiol Group Concentration [µM]	Parameter P	SD	Thiol Group Concentration [µM]
0	0.93	0.04	22.93	0.37	0.05	9.22
50	0.89	0.06	22.12	0.39	0.04	9.56
100	0.92	0.13	22.68	0.49 *	0.05	10.54
150	0.92	0.07	22.71	0.52 *	0.07	10.72

* statistically significant values in relation to controls after 48 h of incubation at *p* < 0.05.

**Table 2:** Catalase (CAT) and peroxidase (GPx) activity converted into an mg of hemoglobin protein in the suspension of erythrocytes incubated with C_60_(OH)_36_ in the concentration range of 0–150 µg/mL for 3 and 48 h at 37 °C. After incubation, the erythrocytes were subjected to hemolysis.

Concentration C_60_(OH)_36_ [µg/mL]	CAT Activity ± SD [U/mgHb]		GPx Activity ± SD [U/mgHb]	
	3 h	48 h	3 h	48 h
0	3.337 ± 0.166	1.662 ± 0.105	0.0169 ± 0.0025	0.0097 ± 0.0021
50	3.781 * ± 0.220	1.280 * ± 0.066	0.0236 * ± 0.0045	0.0139 * ± 0.0020
100	3.701 * ± 0.138	1.277 * ± 0.088	0.0269 * ± 0.0036	0.0127 * ± 0.0022
150	4.146 * ± 0.206	1.384 * ± 0.074	0.0243 * ± 0.0007	0.0124 * ± 0.0003

* statistically significant values in relation to controls after 3 h and 48 h of incubation at *p* <0.05.

**Table 3:** Glutathione reductase and glutathione transferase activity converted to 1 mg of hemoglobin protein in the suspension of erythrocytes incubated with C_60_(OH)_36_ in the concentration range of 0–150 µg/mL for 3 and 48 h at 37 °C. After incubation, the erythrocytes were subjected to hemolysis.

Concentration C_60_(OH)_36_ [µg/mL]	GSR Activity ± SD [U/mgHb]		GST Activity ± SD [U/mgHb]	
	3 h	48 h	3 h	48 h
0	0.0203 ± 0.0027	0.0155 ± 0.0031	0.0506 ± 0.0092	0.0333 ± 0.0052
50	0.0188 ± 0.0039	0.0164 ± 0.0031	0.0331 * ± 0.0080	0.0269 ± 0.0059
100	0.0197 ± 0.0044	0.0202 * ± 0.0046	0.0412 * ± 0.0063	0.0153 * ± 0.0040
150	0.0188 ± 0.0034	0.0210 * ± 0.0036	0.0329 * ± 0.0062	0.0172 * ± 0.0058

* statistically significant values relative to controls after 3 and 48 h incubation at *p* < 0.05.

**Table 4:** Values of *η* determined using Tempamine in a suspension of erythrocytes incubated with C_60_(OH)_36_ in the concentration range of 0–150 µg/mL for 3 and 48 h at 37 °C.

Concentration C_60_(OH)_36_ [µg/mL]	η ± SD * [Pa × s]	
	3 h	48 h
0	4.64 ± 0.57	5.17 ± 0.59
50	5.14 ± 0.38	5.72 ± 0.56
100	4.47 ± 0.29	5.32 ± 0.73
150	4.72 ± 0.99	5.15 ± 0.51

* Results are presented as mean values ± SD of 3–5 independent measurements.

## Data Availability

Not applicable.
